# Immuno-Inflammatory Characteristics in Low Severity COVID-19 Patients with Digestive Symptoms

**DOI:** 10.1155/2020/1063254

**Published:** 2020-08-18

**Authors:** Caihan Duan, Shengyan Zhang, Jian Wang, Wei Qian, Chaoqun Han, Xiaohua Hou

**Affiliations:** Division of Gastroenterology, Union Hospital, Tongji Medical College, Huazhong University of Science and Technology, Wuhan 430022, China

## Abstract

**Aim:**

The outbreak of Coronavirus Disease 2019 (COVID-19) has resulted in a global pandemic, with the main manifestations being of respiratory nature, including pneumonia. It is noteworthy that digestive symptoms are also observed in COVID-19 patients. In this article, we describe the immuno-inflammatory characteristics of low severity COVID-19 patients with digestive symptoms.

**Methods:**

Patients with mild symptoms of COVID-19 were split into three groups based on the patients' symptoms. The first group displayed digestive symptoms only, the second group displayed respiratory symptoms only, and the last group displayed both digestive and respiratory symptoms. Patients were discharged based on negative results of rRT-PCR testing for SARS-CoV-2 from at least two sequential respiratory tract specimens collected ≥24 hours apart. Multiorgan function and immuno-inflammatory characteristics were analyzed for all of the three groups.

**Results:**

Mild liver damage and activation of the immuno-inflammatory system were the most common abnormalities observed in patients with mild COVID-19 symptoms but no significant differences were found (*P* > 0.05). Patients with digestive symptoms were more likely to have slightly higher and later peak values of inflammatory cytokines during the subsequent course of disease (*P* < 0.05). In addition, a significant correlation between IL-2 and TNF level was found in the first group which included patients with digestive symptoms only (*P* < 0.05).

**Conclusions:**

Patients with mild cases of COVID-19 only displaying digestive symptoms are a special subtype. Patients in this group were more likely to have slightly higher and delayed peak values of inflammatory cytokines during the subsequent course of the disease. Prevention and clinical management of this type should be taken into consideration.

## 1. Introduction

Coronavirus disease 2019 (COVID-19) has spread globally and has been declared an international public health emergency by the World Health Organization (WHO) [1, 2]. Respiratory symptoms include coughing, shortness of breath, and dyspnea, which are considered the most common clinical manifestations of COVID-19 [3]. However, recent research has reported additional digestive symptoms which include nausea, vomiting, and diarrhea, which were sometimes the only symptoms for patients [4, 5].

The virus targets the cell receptor Angiotensin-Converting Enzyme 2 (ACE2), which is highly expressed in gastrointestinal organs such as the small intestine and duodenum providing the foundation of gastrointestinal infection [6]. However, viral nucleic acid was also found in feces. 23.29% of patients had positive stool samples after negative respiratory samples, indicating patients who successfully recovered from COVID-19 based on the rRT-PCR testing for respiratory tract specimens may still result in damage of the digestive system [7, 8].

Actually, even though the virus is highly contagious/pandemic, 80.9% to 86.0% of patients were found to experience mild symptoms [9]. More importantly, patients with mild symptoms facilitate the rapid dissemination of 2019-nCoV and are the main cause of the spread of the infection [10]. Therefore, understanding the characteristics of this group could help treat and control the dissemination at an early stage. Our previous study has shown that patients with digestive symptoms are more likely to have a longer delay before viral clearance compared to patients with only respiratory symptoms [11, 12]. However, the underlying reasons remain largely unknown.

In highly pathogenic human coronaviruses (hCoVs), such as severe acute respiratory syndrome CoV (SARS-CoV), and middle east respiratory syndrome CoV (MERS-CoV) infection, cytokine storm and immunosuppression often led to more severe clinical deterioration [13]. Similarly, it has been confirmed that severe COVID-19 cases had markedly higher levels of inflammatory cytokines and a lower absolute number of T lymphocytes [14]. Further research showed SARS-CoV-2 virus infection led to lymphopenia and cytokine storm which correlated with disease severity [15]. Nevertheless, inflammatory and immune characteristics in low severity patients with digestive symptoms remain largely unknown. Therefore, the present study aims at illustrating immuno-inflammatory characteristics and their longitudinal variations in patients with mild COVID-19 symptoms.

## 2. Methods

### 2.1. Patient Diagnosis and Groups

Research was performed at Union Hospital, Tongji Medical College (Wuhan, China), which was a designated hospital for the management of COVID-19 patients. We enrolled 206 patients with mild severity between February 13 and February 29, 2020. Real-Time Reverse-Transcriptase Polymerase-Chain-Reaction (rRT-PCR) was performed on nasal and pharyngeal swabs [16], which were carried out in accordance with the protocol established by the World Health Organization [17]. Patients with mild symptoms were defined as patients without dyspnea, no clinical evidence of respiratory distress, and who were able to maintain blood oxygen saturation above 93% in resting condition [3, 11]. Patients were separated into three groups based on whether they had digestive symptoms such as nausea, vomiting, diarrhea, and anorexia, and/or respiratory symptoms including coughing/expectoration, chest tightness, pharyngalgia, and shortness of breath. The groups were named “Digestive Only,” “Respiratory Only,” and “Digestive+Respiratory”.

The Medical Ethical Review Committee of Union Hospital at Tongji Medical College, Huazhong University of Science and Technology, China, approved this study ([2020] No.0033). The requirement of written informed consent was waived due to the retrospective design of the study and the rapid emergence of COVID-19. Patients' information had been anonymized and de-identified.

### 2.2. Data Collection

In order to obtain more accurate descriptions of the patients' gastrointestinal symptoms, we either conducted a telephone or face to face. To distinguish the reasons of diarrhea, we analyzed the onset date of diarrhea with the start date of any administered drugs that may result in diarrhea such as antiviral therapy. If diarrhea occurred after medication, we removed them from the data. We also interviewed patients who only had respiratory symptoms to ensure they did not have gastrointestinal symptoms too. Epidemiological, clinical manifestations, laboratory, and outcome data were extracted from electronic medical and nursing records. Laboratory examination included routine blood and stool tests, blood chemistry (including electrolytes, liver and renal function, lactate dehydrogenase, and creatine kinase, etc.), coagulation test, inflammatory biomarkers (including C-reactive protein, erythrocyte sedimentation rate (ESR), procalcitonin, IL-2, IL-4, IL-6, IL-10, TNF, etc.), immunity index (including percentage of NK cells, B/CD3^+/^CD4^+/^CD8^+^ lymphocytes, C3, C4, IGE, IGG, IGM, etc.). All data were calculated from the onset date of the disease and checked by two trained doctors. The clinical data were monitored up to March 18, 2020, the final date of follow-up.

### 2.3. Statistical Analysis

Results were presented as numbers and percentages for categorical variables. Continuous variables were described as mean ± standard deviation (SD), mean, maximum, and minimum depending on the situation. Chi-square tests or Fisher exact tests were applied in comparison of categorical variables. Wilcoxon rank sum test and Pearson correlation analysis were applied to continuous variables as appropriate. For the results with the suggested correlation, a scatter graph was applied. Following this, linear regression analysis was carried out to calculate *R*^2^ and *P* values. Statistical analysis was performed using IBM SPSS Statistics version 25.0 (SPSS Inc., IBM Corp., Armonk, NY, USA). Differences were defined as statistically significant at *P* ≤ 0.05 (two-sided).

## 3. Results

### 3.1. General Clinical Characteristics

The entire cohort of 206 patients was living in Wuhan with a median age of 62.5 years (ranged from 27 to 92 years). 89 patients (43.2%) were younger than 60 years, and most patients are retirees (68.9%) and female (55.8%). The most common comorbidity in COVID-19 patients was hypertension (27.2%), diabetes (10.2%), and cardiovascular disease (12.7%). There were 48 patients in the first group, Digestive Only (23.3%), 89 patients in the second group, Respiratory Only (43.2%), and 69 patients in the third group, Digestive+Respiratory (33.5%). A series of respiratory symptoms included cough/expectoration (25.7%), chest tightness (23.8%), shortness of breath (14.6%), and pharyngodynia (6.4%). There were 67 patients (57.3%) who only experienced digestive symptoms who presented diarrhea. Patients who reported diarrhea were more likely to have a fever in the first group who only experienced digestive symptoms (13/23, 56.5% vs. 6/25, 24.0%, *P* = 0.021) and were younger (49.5y vs. 61.4y, *P* = 0.001) in the Digestive+Respiratory group (Table 1).

### 3.2. Mild Liver Damage and Activation of the Immuno-Inflammatory System Are the Most Common Manifestations in Mild Patients upon Admission

Mild liver injury was most commonly observed in mild patients upon admission. In patients with increased ALT (30.6%) and AST (15.0%) levels, the mean value was 36.4 U/L and 31.9 U/L, respectively. Most (17.5%) of the abnormal ALT and AST levels were 40~80 U/L, and those ≥80 U/L were less than 5%. Abnormal LDH was also common, accounting for 32.0%. It seems that the proportion of abnormal liver function in the Respiratory Only group was slightly higher than that of the other two groups but not statistically significant (Table 2, *P* = 0.186). The function of bile secretion also slightly declined with increased levels of TBil and DBil, and increased activities of ALP and GGT. The levels of total protein (TP) and albumin were slightly decreased which indicated that hepatic synthetic function was acceptable. Other abnormal levels of creatinine, electrolyte disturbance, troponin, platelets, coagulation indexes, and D-dimer were not common. Overall, the hepatobiliary system was more vulnerable to damage while renal and cardiac dysfunctions were rare in mild patients.

In patients enrolled in this study, there were 188 (91.3%) patients examined for IL-2, IL-4, IL-6, IL-10, and TNF-*α*. 78 (37.9%) patients were examined for the percentage of NK cells, B lymphocytes. 196 (95.1%) patients were examined for the percentage of CD3+/CD4+/CD8+lymphocytes. 73(35.4%) patients were examined for C3, C4, IGE, IGG, and IGM. With regards to inflammatory cytokines (Table 3), 20 (9.7%) and 69 (33.5%) patients had neutropenia and lymphopenia, respectively. Procalcitonin was normal in all cases. The Respiratory Only group exhibited more enhanced levels of CRP (59.7% vs. 37.2%) and TNF-a (7.4 pg/ml vs. 4.19 pg/ml) than those of the Digestive Only group (all *P* < 0.05). The minority of patients with mild symptoms presented dysregulation of cytokines such as IL-2 (4, 2.1%), IL-4 (15, 8.0%), IL-10 (26, 13.8%), and TNF-*α* (6, 3.2%), while almost everyone showed an increase in IL-6 (178, 94.7%). More importantly, the levels of IL-2 (2.92 pg/ml vs. 2.62 pg/ml, *P* = 0.003), IL-4 (2.57 pg/ml vs. 2.15 pg/ml, *P* = 0.009), and IL-10 (4.42 pg/ml vs. 3.75 pg/ml, *P* = 0.08) were higher in the Digestive Only group compared to the Respiratory Only group. Among groups, no significant differences in immune system parameters were found (*P* > 0.05).

### 3.3. Dynamic Longitudinal Changes of the Immune System in Patients with Mild Symptoms

**Figure 1:**
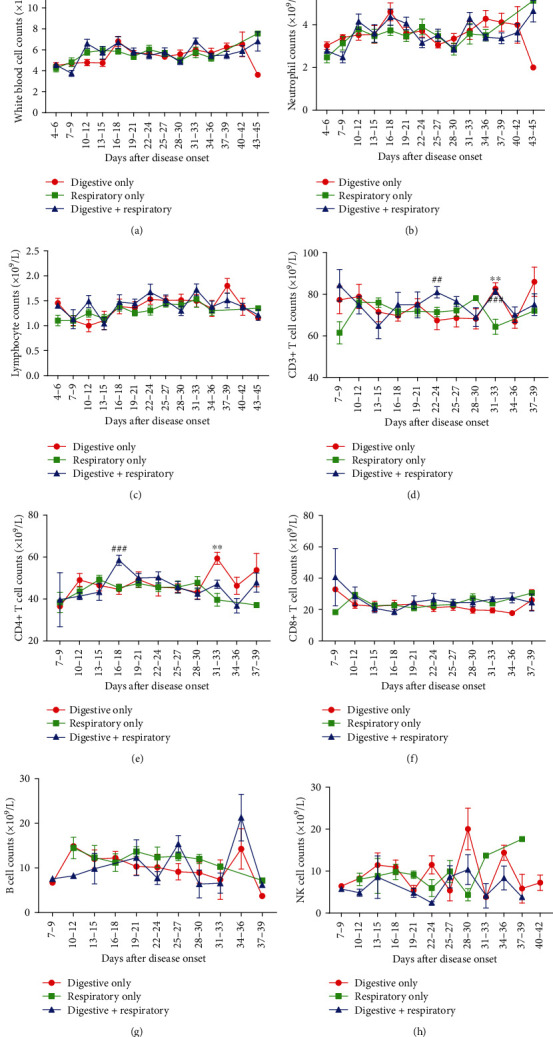
Dynamic profile of peripheral immune cell subsets in COVID-19 patients with different symptoms. Timeline charts illustrate the peripheral WBCs (a), neutrophils (b), lymphocytes (c), CD3^+^ T cell (d), CD4^+^ T cell (e), CD8^+^ T cell (f), B cell (g), and NK cell (h). Error bars, mean ± SEM; ^∗∗^*P* < 0.01, Digestive Only vs. Respiratory Only. ^##^*P* < 0.01, ^###^*P* < 0.001, Digestive+Respiratory vs. Respiratory Only.

**Figure 2:**
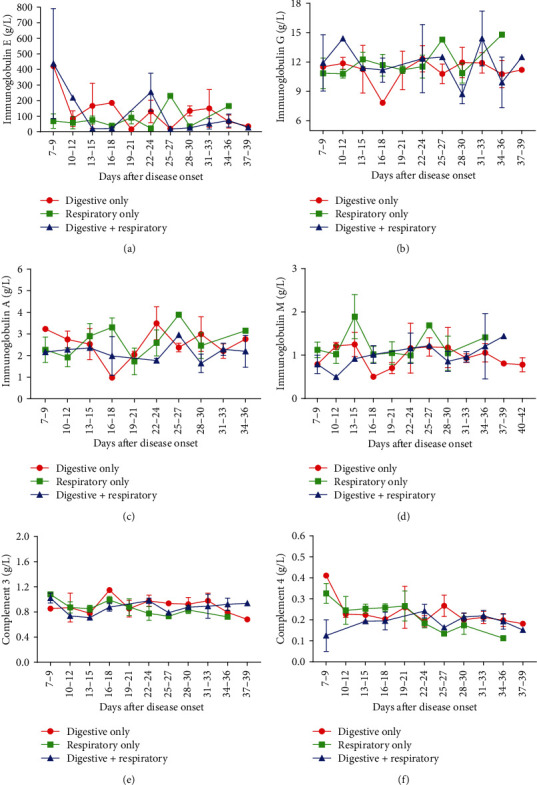
Dynamic profile of immunoglobulin and complement levels in COVID-19 patients with different symptoms. Timeline charts illustrate the levels of IgE (a), IgG (b), IgA (c), IgM (d), C3 (e), and C4 (f). Error bars, mean ± SEM; ^∗∗^*P* < 0.01, Digestive Only vs. Respiratory Only. ^##^*P* < 0.01, ^###^*P* < 0.001.

We then analyzed the dynamic changes of white blood cells (WBCs), neutrophils, lymphocytes, and different lymphocyte subsets in the peripheral blood of patients with mild symptoms. No obvious differences of WBC and neutrophil counts were found between the three groups (Figures 1(a) and 1(b)). Lymphocyte count in the Digestive Only group was higher than Respiratory Only group between 4-6 days but without a statistical significance (1.46 vs. 1.10, *P* = 0.153) and became similar during the subsequent course of the disease (Figure 1(c)). As for the lymphocyte subsets, CD3+ and CD8+ T cell counts of patients with digestive symptoms were higher at 7-9 days (CD3+, 77.32 vs. 61.48 vs. 84.33, *P* = 0.123, CD8+, 32.87 vs. 18.37 vs. 40.64, *P* = 0.375). At 31-33 days, a peak value of CD3+ and CD4+ T lymphocytes appeared in the Digestive Only and the Digestive+Respiratory groups when compared with the Respiratory Only group. (CD3+, 82.75 vs. 64.41, *P* = 0.016, 81.27 vs. 64.41, *P* = 0.0002, CD4+, 59.40 vs. 39.63, *P* = 0.01, 47.06 vs. 39.63, *P* = 0.141, Figures 1(d)–1(f)). We did not observe any significant differences in B cell and NK cell counts during the whole course (Figures 1(g) and 1(h)). The dynamic profile of immunoglobulin and complement levels were also analyzed among the three groups but no significant differences were found (Figure 2).

### 3.4. Dynamic Longitudinal Changes of Inflammatory Cytokines in Patients with Mild Symptoms

**Figure 3:**
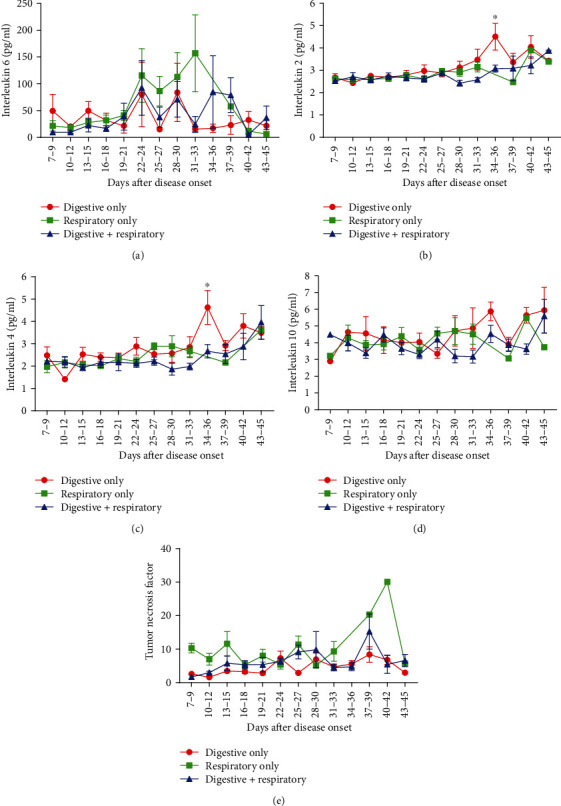
Dynamic profile of peripheral cytokine levels in COVID-19 patients with different symptoms. Timeline charts illustrate the peripheral IL-6 (a), IL-2 (b), IL-4 (c), IL-10 (d), and TNF-*α* (e). Error bars, mean ± SEM; ^∗^*P* < 0.05, Digestive Only vs. Digestive+Respiratory.

We further analyzed the dynamic changes of peripheral IL-2, IL-4, IL-6, IL-10, and TNF-*α* levels. As is shown in Figure 3, IL-6 presented a fluctuation during day 19 to 39, especially in patients with respiratory symptoms only, with a peak at 31-33 days (Figure 3(a)). However, compared with the moderate trend of IL-2, IL-4, IL-10, and TNF-*α* in the Respiratory Only group, a significant increase of IL-2 and IL-4 was seen in the in Digestive Only group at 34-36 days (IL-2, 4.51 vs. 3.08, *P* = 0.049, IL-4, 4.63 vs. 2.67, *P* = 0.041, Figures 3(b)–3(e)).

### 3.5. Significant Linear Correlation between Inflammatory Factors in Digestive Only Patients

**Figure 4:**
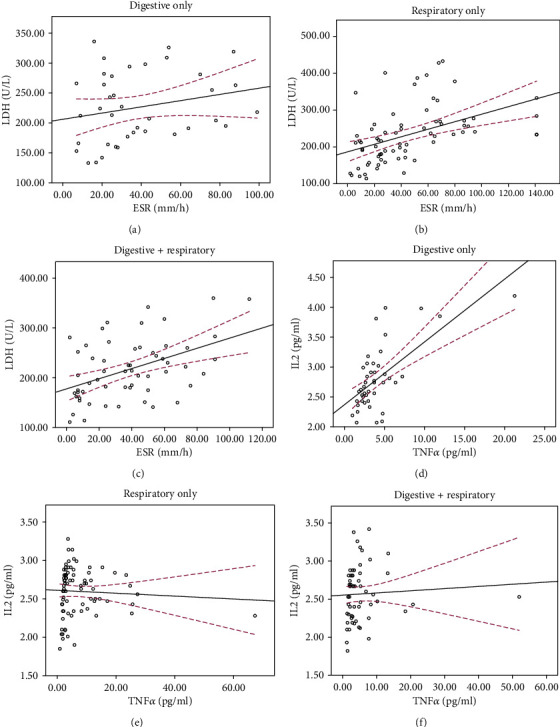
Correlation analysis of IL-2 levels and TNF-*α* levels (a–c), LDH levels and ESR levels (d–f) in COVID-19 patients with different symptoms. The black line is a linear fitting line and the red dotted line represents the confidence interval of the average.

Finally, we used Pearson correlation analysis to compare the correlation between the inflammatory indicators among the three groups. As shown in Figure 4, the level of LDH and ESR in the Respiratory Only group and the Digestive+Respiratory group showed stronger linear correlation (Respiratory Only group: *R*^2^ = 0.211, *R* = 0.459, *P* < 0.001; Digestive+Respiratory group: *R*^2^ = 0.212, *R* = 0.46, *P* < 0.001) than the patients in the Digestive Only group (*R*^2^ = 0.052, *R* = 0.227; *P* = 0.171). However, compared with the Respiratory Only group, the Digestive Only cases showed stronger correlation and linearity in IL-2 and TNF-a (*R*^2^ = 0.003, *R* = −0.057; *P* = 0.615 vs. *R*^2^ = 0.443, *R* = 0.666; *P* < 0.001, respectively).

## 4. Discussion

In the current study, we compared organ function, inflammation, and immune characteristics in subgroups of low severity patients based on whether patients presented digestive symptoms or not. We found that mild liver disorder and the activation of the immuno-inflammatory system were the most common abnormalities in mild patients. Additionally, inflammatory markers showed slightly higher and delayed peak values during the subsequent course of disease in the Digestive Only group compared to the Respiratory Only group.

Liver disorder was the most common abnormality in patients with mild symptoms in our study. Recently, the Lancet also published an article, which reported 43 of the 99 cases (43.4%) of COVID-19 displayed different degrees of liver function damage [9], however, this was found to be more prevalent in critically ill patients. Our study found that the hepatobiliary system was more vulnerable to damage even if the patients had relatively mild symptoms.

Our results further demonstrated that the levels of some inflammatory cytokines such as IL-2, IL-4, and IL-10 in patients with digestive symptoms only were slightly higher compared to those with respiratory symptoms and had delayed peak values during the course of the disease. This might explain why some patients exhibit a longer delay before viral clearance and may be more likely to be associated with poorer prognosis when compared to patients with respiratory symptoms [18].

Compared with the Respiratory Only group, our results demonstrated a significant correlation between IL-2 and TNF level in the Digestive Only group. IL-2 plays a significant role in inducing and enhancing cytotoxic activity [19]. Early studies have described two different patterns of T-cell differentiation which were called “antigen mode” and “inflammation mode.” The expression level of Il-2 varies greatly between these two modes and studies have also found that TNF plays an important role in regulating the conversion of these two modes [20]. Therefore, this phenomenon might suggest that respiratory and digestive symptoms may correspond to two different T-cell differentiation patterns. On the other hand, changing patterns may also be a reason for the different symptoms. This provides a new way for us to further study the pathogenesis and treatment of the disease. The mechanism of gastrointestinal symptoms and its significance still need to be studied further.

Our study has some notable limitations. First, the scale of this study was small and more patients from other regions should have been included to build a more comprehensive understanding of 2019­nCoV and its effect on the digestive system. However, the data in this study provides an early assessment of the inflammatory immune characteristics of gastrointestinal findings in COVID-19 patients. Second, data on critically ill patients was not included. However, the aim at studying immune-inflammatory characteristics in mild cases might be our advantage. Third, not many obvious differences in the longitudinal analysis of immuno-inflammatory characteristics were found. Therefore, further studies, which focus on these aspects are required.

In conclusion, patients with gastrointestinal symptoms only were more likely to have slightly higher and delayed peak values of inflammatory cytokines during the subsequent course of coronavirus disease. The prevention and clinical management of this type should be taken into consideration.

## Figures and Tables

**Table 1:** Clinical characteristics based on the classification of digestive symptoms with or without diarrhea of Digestive Only and Digestive+Respiratory patients on admission.

Items	Total (including Respiratory Only)			Digestive Only			Digestive+Respiratory		
	With diarrhea (*N* = 67)	Without diarrhea (*N* = 139)	*P*	With diarrhea (*N* = 23)	Without diarrhea (*N* = 25)	*P*	With diarrhea (*N* = 44)	Without diarrhea (*N* = 25)	*P*
Age (years)	51.6 (27-84)	62.0 (27-92)	0.128	55.5 (32-84)	67.6 (32-92)	0.424	49.5 (27-81)	61.4 (36-84)	0.001
Gender (male/female)	23/44	68/71	0.048	6/17	7/18	0.882	17/27	13/12	0.282
Fever	48 (71.6%)	90 (64.7%)	0.324	13 (56.5%)	6 (24.0%)	0.021	35 (79.5%)	19 (76.0%)	0.731
Highest temperature, °C	39.8	39.8	-	39	39.8	-	39.8	39.5	-

Organ function injury									
TBil (≤19.0 *μ*mol/L)	7 (10.4%)	10 (7.2%)	0.436	2 (8.7%)	2 (8.0%)	1	5 (11.4%)	2 (8.0%)	1
DBil (≤6.8 *μ*mol/L)	6 (9.1%)	15 (10.9%)	0.696	3 (13.0%)	0 (0.0%)	0.205	3 (7.0%)	2 (8.0%)	1
ALT (≤35 U/L)	17 (25.4%)	54 (39.1%)	0.052	7 (30.4%)	8 (32.0%)	0.907	10 (22.7%)	9 (36.0%)	0.235
AST (≤40 U/L)	6 (9.0%)	25 (18.1%)	0.086	2 (8.7%)	5 (20.0%)	0.484	4 (9.1%)	6 (24.0%)	0.182
ALP (<150 U/L)	0 (0.0%)	5 (3.6%)	0.274	0 (0.0%)	2 (8.0%)	0.508	0 (0.0%)	0 (0.0%)	-
GGT (<50 U/L)	16 (23.9%)	29 (21.0%)	0.642	4 (17.4%)	6 (24.0%)	0.836	12 (27.3%)	3 (12.0%)	0.139
LDH (<245 U/L)	23 (34.3%)	46 (34.1%)	0.971	9 (39.1%)	10 (40.0%)	0.951	14 (31.8%)	8 (32.0%)	0.988
hsTNI (<26.2 ng/L)	0 (0.0%)	4 (3.5%)	0.463	0 (0.0%)	1 (4.8%)	1	0 (0.0%)	0 (0.0%)	-
D-dimer (<0.5 ng/L)	25 (45.5%)	57 (52.3%)	0.408	7 (36.8%)	12 (75.0%)	0.041	18 (50.0%)	11 (50.0%)	1

Immune inflammation									
WBC (3.5-9.5 G/L)	7 (10.4%)	29 (21.2%)	0.059	2 (8.7%)	4 (16.7%)	0.666	5 (11.4%)	6 (24.0%)	0.3
Neutrophil (1.8-6.3 G/L)	7 (10.4%)	28 (20.4%)	0.075	3 (13.0%)	5 (20.8%)	0.701	4 (9.1%)	6 (24.0%)	0.182
Lymphocyte (1.1-3.2 G/L)	18 (26.9%)	52 (38.0%)	0.117	6 (26.1%)	10 (41.7%)	0.260	12 (27.3%)	7 (28.0%)	0.948
CRP (<8 mg/L)	21 (38.9%)	66 (54.5%)	0.056	7 (36.8%)	9 (37.5%)	0.965	14 (40.0%)	9 (45.0%)	0.718
ESR (<15 mm/h)	40 (71.4%)	103 (84.4%)	0.043	14 (77.8%)	20 (90.9%)	0.381	26 (68.4%)	19 (86.4%)	0.122
IL-2 (0.1-4.1 pg/ml)	3 (4.8%)	1 (0.8%)	0.214	3 (13.0%)	1 (4.2%)	0.348	0 (0.0%)	0 (0.0%)	-
IL-4 (0.1-3.2 pg/ml)	4 (6.3%)	11 (8.8%)	0.558	2 (8.7%)	5 (20.8%)	0.416	2 (5.0%)	1 (5.0%)	1
IL-6 (0.1-2.9 pg/ml)	59 (93.7%)	119 (95.2%)	0.918	20 (87.0%)	23 (95.8%)	0.348	39 (97.5%)	18 (90.0%)	0.255
IL-10 (0.1-5.0 pg/ml)	6 (9.5%)	20 (16.0%)	0.225	3 (13.0%)	8 (33.3%)	0.101	3 (7.5%)	2 (10.0%)	1
TNF-a (0.1-23 pg/ml)	0 (0.0%)	6 (4.8%)	0.184	0 (0.0%)	0 (0.0%)	-	0 (0.0%)	0 (0.0%)	-
IgG (7.51-15.6 g/L)	3 (13.0%)	3 (6.0%)	0.576	2 (15.4%)	1 (6.7%)	0.583	1 (10.0%)	1 (25.0%)	0.505
IgM (0.46-3.04 g/L)	1 (4.3%)	4 (8.0%)	0.940	0 (0.0%)	0 (0.0%)	-	1 (10.0%)	0 (0.0%)	1
C3 (0.79-1.52 g/L)	3 (13.0%)	14 (28.0%)	0.160	2 (15.4%)	4 (26.7%)	0.655	1 (10.0%)	1 (25.0%)	0.505
C4 (0.16-0.38 g/L)	3 (13.0%)	13 (26.0%)	0.214	0 (0.0%)	4 (26.7%)	0.102	3 (30.0%)	2 (50.0%)	0.580
CD3+T lymphocytes	19 (30.6%)	30 (22.4%)	0.214	7 (31.8%)	8 (33.3%)	0.913	12 (30.0%)	7 (29.2%)	0.944
CD4+T lymphocytes	21 (33.9%)	43(32.1%)	0.805	8 (36.4%)	9 (37.5%)	0.936	13 (32.5%)	7 (29.2%)	0.781
CD8+T lymphocytes	12 (19.4%)	26 (19.4%)	0.994	3 (13.6%)	5 (20.8%)	0.702	9 (22.5%)	6 (25.0%)	0.819

Indicators of organ function injury and immune inflammation were expressed in terms of the number and percentage of abnormalities; Digestive Only: gastrointestinal symptoms cases; Respiratory Only: respiratory symptoms cases; Digestive+Respiratory: both digestive and respiratory symptoms. Because not all patients have the same test, the denominators of each indicator may be inconsistent in their calculations. A statistical test proved that the deletions belong to random deletions and have no influence on the results (Supplementary Table 1). A significance level of *P* ≤ 0.05 was used.

**Table 2:** Multiorgan function change characteristics based on the classification of the main symptoms in COVID-19 patients on admission.

Items	Total (*N* = 206)	Digestive Only(*N* = 48)	Respiratory Only(*N* = 89)	Digestive+Respiratory (*N* = 69)	*P*
Blood biochemistry					
TBil (*μ*mol/L)	12.9 (2.15-35.7)	12.1 (6.7-22.9)	13.1 (2.15-35.7)	13.2 (4.1-26.3)	0.391
≤19.0 *μ*mol/L	189 (91.7%)	44 (91.7%)	83 (93.3%)	62 (89.9%)	0.743
>19.0 *μ*mol/L	17 (8.3%)	4 (8.3%)	6 (6.7%)	7 (10.1%)	
DBil (*μ*mol/L)	4.6 (1.7-16.6)	4.2 (2.3-9.6)	5.1 (2.0-16.6)	4.2 (1.7-9.7)	0.008
≤6.8 *μ*mol/L	185 (89.8%)	45 (93.8%)	76 (85.4%)	64 (92.8%)	0.186
>6.8 *μ*mol/L	21 (10.2%)	3 (6.2%)	13 (14.6%)	5 (7.2%)	
TP (g/L)	63.5 (52.7-81.8)	63.9 (54.2-74.5)	62.9 (58.8-81.8)	64.0 (52.7-76.4)	0.525
<64 g/L	107 (51.9%)	24 (50.0%)	47 (52.8%)	36 (52.2%)	0.951
≥64 g/L	99 (48.1%)	24 (50.0%)	42 (47.2%)	33 (47.8%)	
Albumin (g/L)	36.5 (23.9-68.0)	36.3 (27.8-43.8)	36.3 (24.6-68.0)	36.9 (23.9-47.8)	0.723
<35 g/L	76 (36.9%)	13 (27.1%)	37 (41.6%)	26 (37.7%)	0.242
≥35 g/L	130 (63.1%)	35 (72.9%)	52 (58.4%)	43 (62.3%)	
ALT (U/L)	36.4 (7-498)	32.4 (7-176)	40.8 (7-498)	33.7 (7-152)	0.229
≤35 U/L	143 (69.4%)	35 (72.9%)	60 (67.4%)	48 (69.6%)	0.800
>35 U/L	63 (30.6%)	13 (27.1%)	29 (32.6%)	21 (30.4%)	
AST (U/L)	31.9 (10-346)	29.9 (11-81)	35.4 (12-346)	28.9 (10-135)	0.167
≤40 U/L	175 (85.0%)	41 (85.4%)	75 (84.3%)	59 (85.5%)	0.972
>40 U/L	31 (15.0%)	7 (14.6%)	14 (15.7%)	10 (14.5%)	
ALP (U/L)	72.5 (19-250)	76.4 (27-214)	75.1 (19-250)	66.3 (33-141)	0.100
<150 U/L	201 (97.6%)	46 (95.8%)	86 (96.6%)	69 (100.0%)	0.123
>150 U/L	5 (2.4%)	2 (4.2%)	3 (3.4%)	0 (0.0%)	
GGT (U/L)	38.6 (7-291)	39.6 (8-196)	39.9 (7-291)	36.1 (11-162)	0.786
<50 U/L	157 (76.2%)	39 (81.2%)	65 (73.0%)	53 (76.8%)	0.554
>50 U/L	49 (23.8%)	9 (18.8%)	24 (27.0%)	16 (23.2%)	
LDH (U/L)	230.7 (15-649)	241.2 (88-649)	230.4 (15-527)	223.7 (114-497)	0.549
<245 U/L	140 (68.0%)	29 (60.4%)	60 (67.4%)	48 (69.6%)	0.539
>245 U/L	66 (32.0%)	19 (39.6%)	29 (32.6%)	21 (30.4%)	
CREA (44-133 *μ*mol/L)	73.3 (39.6-430)	67.8 (44.4-121)	79.2 (41.6-430)	69.5 (39.6-121)	0.128
Glu (3.9-6.1 mmol/L)	6.1 (1.79-20.06)	5.62 (3.95-13.49)	6.4 (1.79-15.04)	6.1 (3.94-20.06)	0.700
K (3.5-5.2 mmol/L)	4.0 (2.8-5.79)	4.1 (3.27-5.79)	4.0 (2.8-5.79)	4.0 (2.8-5.1)	0.276
Ca (2.03-2.54 mmol/L)	2.2 (0.2-4.51)	2.2 (1.92-3.21)	2.2 (1.78-4.51)	2.2 (0.2-2.54)	0.642
hsTNI (<26.2 ng/L)	4.6 (0.4-38.0)	4.9 (0.5-34.7)	5.1 (0.4-38.0)	3.5 (0.7-19.2)	0.285
Blood routine and coagulation function					
HGB (130-175 g/L)	122.3 (80-159)	119.6 (94-151)	122.5 (80-161)	123.9 (93-159)	0.390
PLT (125-350 G/L)	241.9 (24-537)	239.9 (91-479)	242.9 (24-463)	241.8 (102-537)	0.982
APTT (28-43.5 s)	37.1 (13.7-56.3)	37.3 (28.1-55.8)	37.7 (26.2-56.3)	36.3 (13.7-49.9)	0.329
PT (11-16 s)	13.3 (11.4-16.6)	13.4 (11.4-15.4)	13.4 (11.9-16.6)	13.2 (11.7-15.0)	0.237
D-dimer (<0.5 ng/L)	1.0 (0.2-20)	1.0 (0.2-20)	1.0 (0.2-13.01)	0.9 (0.2-8.78)	0.383

**Table 3:** Inflammation and immune characteristics based on the classification of main symptoms in COVID-19 patients on admission.

Items	Total	Digestive Only	Respiratory Only	Digestive+Respiratory	*P*
WBC (G/L)	5.54 (1.73-11.64)	5.65 (2.66-11.64)	5.44 (1.73-10.24)	5.58 (2.42-10.48)	0.789
<9.5 G/L	196 (95.1%)	46 (95.8%)	84 (94.4%)	66 (95.7%)	0.906
>9.5 G/L	10 (4.9%)	2(4.2%)	5 (5.6%)	3 (4.3%)	
Neutrophil (G/L)	3.58 (0.93-8.67)	3.61 (1.36-8.1)	3.51 (0.93-8.56)	3.65 (1.13-8.67)	0.854
<6.3 G/L	191 (92.7%)	45 (93.7%)	82 (92.1%)	64 (92.8%)	0.941
>6.3 G/L	15 (7.3%)	3 (6.3%)	7 (7.9%)	5 (7.2%)	
Lymphocyte (G/L)	1.35 (0.28-3.4)	1.41 (0.42-2.18)	1.28 (0.28-3.4)	1.39 (0.39-2.76)	0.302
<1.1 G/L	69 (33.5%)	16 (33.3%)	34 (38.2%)	19 (27.5%)	0.371
≥1.1 G/L	137 (66.5%)	32 (66.7%)	55 (61.8%)	50 (72.5%)	
Procalcitonin (ug/L)	0.14 (0.03-2.99)	0.13 (0.03-2.99)	0.14 (0.03-2.99)	0.14 (0.03-2.99)	0.139
<0.5 ug/L	183 (88.8%)	42 (87.5%)	80 (89.9%)	61 (88.4%)	0.906
≥0.5 ug/L	23 (11.2%)	6 (12.5%)	9 (10.1%)	8 (11.6%)	
C-reactive protein (mg/L)	20.5 (0.3-173)	19.3 (0.6-173)	26.2 (0.3-136)	21.8 (0.6-168)	0.569
<8 mg/L	90 (51.4%)	27 (62.8%)	31 (40.3%)	32 (58.2%)	0.029
≥8 mg/L	85 (48.6%)	16 (37.2%)	46 (59.7%)	23 (41.8%)	
ESR (mm/h)	42.1 (2-140)	38.2 (7-99)	46.8 (2-140)	38.5 (2-112)	0.357
<15 mm/h	35 (19.7%)	6 (15.0%)	14 (17.9%)	15 (25.0%)	0.411
≥15 mg/L	143 (80.3%)	34 (85.0%)	64 (82.1%)	45 (75.0%)	
Inflammatory cytokines					
IL-2 (0.1-4.1 pg/ml)	2.66 (2.07-4.93)	2.92 (2.07-4.93)	2.62 (1.85-4.08)	2.59 (1.82-3.98)	<0.001
IL-4 (0.1-3.2 pg/ml)	2.32 (0.98-8.23)	2.57 (1.04-8.23)	2.15 (1.00-4.18)	2.00 (0.98-4.80)	0.002
IL-6 (0.1-2.9 pg/ml)	40.09 (1.96-437.81)	45.53 (1.96-437.81)	43.18 (2.27-424.93)	31.64 (2.05-383.95)	0.559
<2.9 pg/ml	10 (5.3%)	4 (8.5%)	3 (3.7%)	3 (5.0%)	0.610
2.9-90 pg/ml	159 (84.6%)	37 (78.7%)	69 (85.2%)	53 (88.3%)	
≥90 pg/ml	19 (10.1%)	6 (12.8%)	9 (11.1%)	4 (6.7%)	
IL-10 (0.1-5.0 pg/ml)	3.84 (1.75-15.98)	4.42 (2.15-15.98)	3.75 (1.92-9.34)	3.46 (1.75-6.30)	0.021
TNF-a (0.1-23 pg/ml)	5.76 (0.90-67.26)	4.19 (1.01-21.26)	7.40 (0.90-67.26)	4.78 (1.27-20.73)	0.014
Immune system indexes					
IgG (7.51-15.6 g/L)	11.53 (7.3-16.8)	11.39 (7.30-16.80)	11.67 (8.15-16.60)	11.56 (6.83-19.30)	0.912
IgM (0.46-3.04 g/L)	1.32 (23.8%)	1.06 (0.50-2.31)	1.68 (0.39-12.03)	1.03 (0.46-1.96)	0.200
C3 (0.79-1.52 g/L)	0.91 (14.6%)	0.94 (0.64-1.24)	0.90 (0.53-1.24)	0.91 (0.71-1.10)	0.684
C4 (0.16-0.38 g/L)	0.23 (6.4%)	0.23 (0.14-0.41)	0.24 (0.08-0.41)	0.19 (0.03-0.29)	0.055
CD3+T lymphocytes (58.17-84.22%)	73.07 (26.12-93.02)	73.67 (45.44-93.02)	72.12 (49.40-85.88)	73.10 (26.12-92.20)	0.996
CD4+T lymphocytes (25.34-54.37%)	46.69 (10.70-75.73)	48.87 (24.19-75.73)	47.62 (35.22-64.48)	44.67 (10.70-70.46)	0.186
CD8+T lymphocytes (14.23-38.95%)	22.47 (5.09-58.88)	21.40 (7.57-38.74)	21.33 (7.27-34.99)	23.78 (5.09-58.88)	0.282

## Data Availability

All data generated or analyzed during this study are included in this article.

## Supplementary Materials

Supplementary Materials Supplementary Table: missing data based on the sample size of digestive symptoms with or without diarrhea of Digestive Only group and Digestive+Respiratory group.
