# Supplementary material for: Immuno-Inflammatory Characteristics in Low Severity COVID-19 Patients with Digestive Symptoms
Source: Gastroenterol Res Pract. 2020 Aug 18;2020:1063254. doi: 10.1155/2020/1063254 (PMC7450334; doi:10.1155/2020/1063254)
Supplement: Supplementary Materials — Supplementary Table: missing data based on the sample size of digestive symptoms with or without diarrhea of Digestive Only group and Digestive+Respiratory group. [file 1063254.f1.pdf]

Supplementary Table. missing data based on the sample size of digestive symptoms with or without diarrhea of Digestive only group and Digestive+Respiratory group.

Items	Total(including Respiratory only)			Digestive only			Digestive+Respiratory		
	With Diarrhea (N=67)	Without Diarrhea (N=139)	P	With Diarrhea (N=23)	Without Diarrhea (N=25)	P	With Diarrhea (N=44)	Without Diarrhea (N=25)	P
**Gender (Male/Female)**	0	0		0	0		0	0	
**Fever**	0	0		0	0		0	0	
**Organ function injury**									
**TBil**	0	0		0	0		0	0	
**DBil**	0	0		0	0		0	0	
**ALT**	0	1	1	0	0		0	0	
**AST**	0	1	1	0	0		0	0	
**ALP**	0	0		0	0		0	0	-
**GGT**	0	0		0	0		0	0	
**LDH**	0	4	0.306	0	0		0	0	
**hsTNI**	12	25	0.989	3	4	1	4	3	0.698
**D-Dimer**	12	30	0.540	4	9	0.147	8	3	0.740
**Immune inflammation**									
**WBC**	0	2	1	0	1	**1**	0	0	
**Neutrophil**	0	2	1	0	1	1	0	0	
**Lymphocyte**	0	2	1	0	1	1	0	0	
**CRP**	13	18	0.225	4	1	0.180	9	5	0.964
**ESR**	11	17	0.411	5	3	0.454	6	3	1
**IL-2**	4	14	0.329	0	1	1	4	5	0.357
**IL-4**	4	14	0.329	0	1	1	4	5	0.357
**IL-6**	4	14	0.329	0	1	1	4	5	0.357
**IL-10**	4	14	0.329	0	1	1	4	5	0.357
**TNF-a**	4	14	0.329	0	1	1	4	5	0.357
**IgG**	44	89	0.817	10	10	0.807	34	21	0.504
**IgM**	44	89	0.817	10	10	0.807	34	21	0.504
**C3**	44	89	0.817	10	10	0.807	34	21	0.504
**C4**	44	89	0.817	10	10	0.807	34	21	0.504
**CD3+Tlymphocytes**	5	5	0.388	1	1	1	4	1	0.646
**CD4+ T lymphocytes**	5	5	0.388	1	1	1	4	1	0.646
**CD8+ T lymphocytes**	5	5	0.388	1	1	1	4	1	0.646
